# Quercetin protects HTR-8/SVneo human trophoblast cells against oxidative stress injury via activating EGFR/PI3K/AKT-NRF2/HO-1 signaling axis

**DOI:** 10.3389/fphar.2026.1887502

**Published:** 2026-08-06

**Authors:** Lujia Liu, Zhixian Ding, Yina Wu, Jianxiong Xiao, Caixia Tao, Xiaoyan Zhou, Heng Tang

**Affiliations:** 1 Key Laboratory of Reproductive Medicine and Embryo, Wanbei Coal Electric Group General Hospital, Suzhou, Anhui, China; 2 Central Laboratory, Wanbei Coal Electric Group General Hospital, Suzhou, Anhui, China; 3 Intensive Care Unit, Wanbei Coal Electric Group General Hospital, Suzhou, Anhui, China

**Keywords:** EGFR/PI3K/AKT, HTR-8/SVneo cell, NRF2/HO-1, oxidative stress, quercetin

## Abstract

Adverse pregnancy outcomes represented by recurrent spontaneous abortion remain a global clinical problem, with excessive oxidative stress-induced trophoblast dysfunction as its well-recognized core pathological mechanism. Quercetin is a natural flavonoid with strong antioxidant and cytoprotective effects, but its protective effect and molecular mechanism on human trophoblasts under oxidative stress remain unclear. This study investigated whether quercetin alleviates H_2_O_2_-induced oxidative damage in HTR-8/SVneo trophoblasts via regulating the EGFR/PI3K/AKT-NRF2/HO-1 axis. We performed CCK-8 assay, flow cytometry, migration assays, RT-qPCR, Western blot, molecular docking and network pharmacology. The results showed that quercetin pretreatment significantly improved the viability of injured trophoblasts, inhibited apoptosis, alleviated G0/G1 phase arrest, restored migration, maintained redox homeostasis, and upregulated NRF2 and HO-1. Mechanistically, computational predictions suggest that quercetin may interact with EGFR and enhance EGFR/PI3K/AKT phosphorylation; inhibition of EGFR or NRF2 significantly reversed the protective effects of quercetin. In conclusion, quercetin protects trophoblasts against oxidative stress injury via activating the EGFR/PI3K/AKT-NRF2/HO-1 axis, providing a novel mechanism and potential intervention for adverse pregnancy outcomes.

## 1 Introduction

Adverse pregnancy outcomes (APOs), including preeclampsia (PE), fetal growth restriction (FGR), and unexplained recurrent spontaneous abortion (URSA), are the leading causes of maternal and perinatal morbidity and mortality worldwide, imposing a heavy burden on the global public health system (Li et al., 2025; Wang C. et al., 2025). The establishment and maintenance of a successful pregnancy are highly dependent on normal placental development. The proliferation, cell cycle homeostasis, anti-apoptotic capacity, and migration and invasion functions of extravillous trophoblasts serve as the core biological basis for uterine spiral artery remodeling and sufficient placental perfusion (DeMayo, 2024; Jameel et al., 2024). Accumulating evidence has confirmed that trophoblast dysfunction mediated by excessive oxidative stress in the placental microenvironment is the core pathological mechanism underlying the onset of the aforementioned APOs (Wang R et al., 2025; Zhang et al., 2024). At present, there is still a lack of safe and effective targeted prevention and treatment strategies for oxidative stress-related placenta-derived APOs in clinical practice. Therefore, screening bioactive compounds that can alleviate oxidative stress-induced trophoblast injury has become a critical research direction in obstetrics.

Quercetin(Que), a natural flavonoid widely present in vegetables, fruits, and medicinal plants, has been well-documented to possess robust antioxidant, anti-inflammatory, anti-apoptotic, and organ-protective pharmacological activities (Alharbi et al., 2025; Lin et al., 2025). Existing studies have shown that quercetin can attenuate oxidative stress injury in various cell and disease models by regulating the endogenous antioxidant defense system (Ding et al., 2024; Zhang et al., 2025). In the field of reproductive medicine, preliminary studies have verified that quercetin can improve ovarian function and alleviate pregnancy-related complications (Qi L. et al., 2025; Wang et al., 2024). However, to date, the protective effect of quercetin against oxidative stress injury in human trophoblasts has not been fully elucidated, and the core molecular targets and upstream/downstream regulatory networks underlying its antioxidant effect remain unclear, which greatly limits its translational application in obstetrics.

Nuclear factor erythroid 2-related factor 2 (NRF2) is the master regulator of the cellular endogenous antioxidant defense system. It undergoes nuclear translocation, binds to the antioxidant response element (ARE), and initiates the transcription of downstream antioxidant enzymes including heme oxygenase-1 (HO-1), thereby scavenging excess reactive oxygen species (ROS) and maintaining cellular redox homeostasis (Saha et al., 2020; Sethi et al., 2025). Accumulating studies have confirmed that activation of the NRF2/HO-1 signaling pathway significantly alleviates oxidative stress injury in trophoblasts, while the upstream regulatory mechanism of this axis remains to be clarified (Dong et al., 2024; He et al., 2023). The Epidermal Growth Factor Receptor (EGFR)/Phosphatidylinositol 3-Kinase (PI3K)/Protein Kinase B (AKT) axis is a canonical cascade pathway that regulates cell survival, migration, and antioxidant stress response. Existing studies have verified that this pathway modulates the nuclear translocation and transcriptional activity of NRF2 via phosphorylation modification (Kowalczyk et al., 2024; Ni et al., 2026). In our preliminary work, EGFR, PI3K, and AKT were screened as the core functional targets of quercetin through network pharmacology analysis. Molecular docking results further suggested that quercetin may have favorable binding affinity with EGFR, PI3K, AKT, NRF2, and HO-1. Based on these findings, we propose the scientific hypothesis that quercetin protects the biological functions of human trophoblasts by activating the EGFR/PI3K/AKT signaling pathway, which further upregulates the expression of the NRF2/HO-1 antioxidant axis to suppress oxidative stress injury.

In this study, we used the human extravillous trophoblast cell line HTR-8/SVneo as the *in vitro* model, and established a trophoblast oxidative stress injury model with hydrogen peroxide (H_2_O_2_). First, we intervened with gradient concentrations of quercetin, with N-acetylcysteine (NAC) as the positive control, to clarify the protective effect of quercetin on oxidatively injured trophoblasts via detecting cell cycle distribution, apoptosis, migration ability, and core oxidative stress markers (ROS, MDA, and SOD). Subsequently, we verified the regulatory effect of quercetin on the EGFR/PI3K/AKT signaling pathway and the NRF2/HO-1 axis. Finally, we performed inhibitor-mediated reverse validation using the EGFR inhibitor Gefitinib and the NRF2 inhibitor ML385, to clarify the upstream and downstream cascade regulatory relationship between the two pathways, and to elucidate the core molecular mechanism underlying the trophoblast-protective effect of quercetin. This study aims to provide a novel theoretical basis and potential drug candidate for the prevention and treatment of placenta-derived adverse pregnancy outcomes.

## 2 Materials and methods

### 2.1 Cell culture and reagents

#### 2.1.1 Cell culture

The human extravillous trophoblast cell line HTR-8/SVneo was purchased from the Cell Bank of the Chinese Academy of Sciences (Shanghai, China). Cells were cultured in RPMI 1640 medium supplemented with 10% fetal bovine serum and 1% penicillin-streptomycin, and incubated in a humidified incubator at 37 °C with 5% CO_2_. All subsequent experiments were performed using cells in the logarithmic growth phase with good morphology.

#### 2.1.2 Main reagents

Quercetin, hydrogen peroxide (H_2_O_2_), N-acetylcysteine (NAC), Gefitinib (EGFR inhibitor), and ML385 (NRF2 inhibitor) were purchased from MedChemExpress (MCE, United States). Cell Counting Kit-8 (CCK-8) was purchased from Solarbio (China). Annexin V-FITC/PI apoptosis detection kit, cell cycle detection kit, reactive oxygen species (ROS) detection kit, RIPA lysis buffer, BCA protein quantification kit, glutathione (GSH) detection kit, malondialdehyde (MDA) detection kit, and superoxide dismutase (SOD) detection kit were purchased from Beyotime Biotechnology (China). Reverse transcription kit and SYBR Green quantitative real-time PCR (RT-qPCR) kit were purchased from TaKaRa (Japan). Primary antibodies against p-EGFR, EGFR, PI3K, p-PI3K, AKT, p-AKT, NRF2, HO-1 and β-actin were purchased from Cell Signaling Technology (USA). Horseradish Peroxidase (HRP)-conjugated goat anti-rabbit IgG secondary antibody was purchased from Zhongshan Golden Bridge Biotechnology (China).

### 2.2 Network pharmacology analysis

Network pharmacology was applied to screen the core targets and regulatory pathways of quercetin against trophoblast oxidative stress injury, and to provide theoretical direction for subsequent mechanism validation.

#### 2.2.1 Prediction of quercetin targets and disease-related targets

The chemical structure of quercetin was retrieved from the PubChem database. Potential targets of quercetin were predicted using the SwissTargetPrediction, PharmMapper and TCMSP databases. Duplicate and invalid targets were removed, and gene names were standardized through the Uniprot database. Disease-related targets were collected from the GeneCards, OMIM and DisGeNET databases with the keywords of “recurrent spontaneous abortion”.

#### 2.2.2 Construction of drug-disease common targets and PPI network

The intersecting targets between quercetin-related targets and disease-related targets were obtained using Venny 2.1, which were identified as potential therapeutic targets of quercetin against trophoblast injury. The intersecting targets were imported into the STRING database, with the organism set to “*Homo sapiens*” and the minimum interaction confidence score set to ≥0.7. Isolated nodes were excluded to construct the protein-protein interaction (PPI) network. The network was visualized and analyzed using Cytoscape 3.9.1 software, and core targets were screened according to the degree value, betweenness centrality and closeness centrality calculated by the CytoNCA plugin.

#### 2.2.3 GO and KEGG enrichment analysis

The common targets of quercetin and disease were imported into the DAVID database for Gene Ontology (GO) functional enrichment and Kyoto Encyclopedia of Genes and Genomes (KEGG) pathway enrichment analysis, with the organism set to “*Homo sapiens*”, P-value < 0.05 and Q-value < 0.05. The top 10 biological process terms and top 20 signaling pathways were selected for visualization analysis.

### 2.3 Molecular docking simulation

Molecular docking was performed to predict the binding potential and interaction mode between quercetin and the core targets screened by network pharmacology.The 2D structure of quercetin was downloaded from the PubChem database, and its 3D structure was constructed and optimized by energy minimization using ChemBio 3D software, then saved in SDF format. The crystal structures of core targets were retrieved from the RCSB PDB database, Water molecules, original ligands and non-specific subunits were removed from the protein structures using PyMOL software, and polar hydrogens and Gasteiger charges were added via AutoDock Tools 1.5.7 software.Molecular docking was performed using AutoDock Vina 1.2.3 software. The docking grid box was set to cover the active pocket of each target protein, the ligand was set to fully flexible and the receptor backbone was set to rigid, with other parameters kept as default. The conformation with the lowest binding free energy was selected for further analysis, and the binding mode, hydrogen bond interaction and hydrophobic interaction between quercetin and core targets were visualized using PyMOL software. A binding free energy ≤ −5.0 kcal/mol was defined as good binding activity.

### 2.4 Molecular dynamics simulation

All-atom molecular dynamics (MD) simulation was performed using YASARA software to verify the binding stability of the quercetin-EGFR complex. The optimal binding conformation of quercetin and EGFR (PDB ID: 1M14) obtained from molecular docking was used as the initial simulation structure. The AMBER14SB force field was applied for the EGFR protein, and the GAFF2 force field was assigned to the ligand quercetin. The complex was solvated in a periodic cubic box with the TIP3P water model, and Na^+^/Cl^−^ ions were added to neutralize the system charge and maintain a physiological ionic strength of 0.15 M.

The system was first subjected to energy minimization via steepest descent combined with conjugate gradient algorithm to eliminate steric clashes and unreasonable conformations. Two-stage equilibrium simulations were then performed: 500 ps NVT ensemble simulation at 300 K to stabilize the system temperature, followed by 1 ns NPT ensemble simulation at 300 K and 1 bar to balance the system pressure and density. Finally, a 100 ns all-atom production MD simulation was carried out under the NPT ensemble with a time step of 2 fs, and the trajectory was saved every 100 ps for subsequent analysis. The root mean square deviation (RMSD), root mean square fluctuation (RMSF) and MM-PBSA binding free energy of the complex were calculated using the built-in analysis module of YASARA to evaluate the binding stability and affinity of quercetin to EGFR.

### 2.5 Cell viability assay

Cell viability was assessed using the CCK-8 assay to screen the optimal working concentrations of H_2_O_2_, quercetin, NAC, Gefitinib and ML385. HTR-8/SVneo cells were seeded into 96-well plates at a density of 5 × 10^3^ cells/well and cultured overnight for adherence.

Screening of H_2_O_2_ modeling concentration: Cells were treated with gradient concentrations of H_2_O_2_ for 24 h. After treatment, 10 μL of CCK-8 reagent was added to each well and incubated at 37 °C for 2 h. The optical density (OD) value at 450 nm was measured using a microplate reader. The concentration of H_2_O_2_ that reduced cell viability by 40%–50% was selected as the optimal modeling concentration for subsequent experiments.

Screening of safe and protective concentrations of quercetin: For safety evaluation, normal HTR-8/SVneo cells were treated with gradient concentrations of quercetin for 24 h, and cell viability was detected to determine the non-cytotoxic concentration range of quercetin. For protective effect evaluation, cells were pretreated with gradient concentrations of quercetin within the safe range for 2 h, then co-treated with the optimal modeling concentration of H_2_O_2_ for 24 h. Cell viability was detected, and the low and high concentrations of quercetin with significant protective effect were selected for subsequent experiments.

Screening of working concentrations of NAC, Gefitinib and ML385: Cells were treated with gradient concentrations of NAC, Gefitinib and ML385 respectively for 24 h, and cell viability was detected by CCK-8 assay. The maximum non-cytotoxic concentration with stable pharmacological effect was selected as the working concentration for subsequent experiments. The pretreatment time of inhibitors (2 h) was determined by pre-experiments. All experiments were repeated independently at least 3 times.

### 2.6 Cell treatment and experimental groups

HTR-8/SVneo cells in the logarithmic growth phase were randomly divided into the following groups, with 3 replicate wells set for each group in each experiment, and all experiments were repeated independently at least 3 times.

#### 2.6.1 Experiment 1: protective effect of quercetin on H_2_O_2_-Induced trophoblast injury

Control group: Cells were cultured with complete medium only, without any drug intervention;

H_2_O_2_ model group: Cells were treated with the optimal modeling concentration of H_2_O_2_ for 24 h;

Quercetin low-dose group: Cells were pretreated with the selected low concentration of quercetin for 2 h, then co-treated with H_2_O_2_ for 24 h;

Quercetin high-dose group: Cells were pretreated with the selected high concentration of quercetin for 2 h, then co-treated with H_2_O_2_ for 24 h;

NAC positive control group: Cells were pretreated with the selected working concentration of NAC for 2 h, then co-treated with H_2_O_2_ for 24 h.

#### 2.6.2 Experiment 2: inhibitor reverse validation for pathway regulatory relationship

Control group: Cells were cultured with complete medium only;

H_2_O_2_ model group: Cells were treated with the optimal modeling concentration of H_2_O_2_ for 24 h;

H_2_O_2_ + quercetin high-dose group: Cells were pretreated with high-dose quercetin for 2 h, then co-treated with H_2_O_2_ for 24 h;

H_2_O_2_ + quercetin high-dose + gefitinib group: Cells were pretreated with the working concentration of Gefitinib for 2 h, followed by the same treatment as the H_2_O_2_ + quercetin high-dose group;

H_2_O_2_ + quercetin high-dose + ML385 group: Cells were pretreated with the working concentration of ML385 for 2 h, followed by the same treatment as the H_2_O_2_ + quercetin high-dose group.

### 2.7 Wound healing assay

Wound healing assay was performed to detect the horizontal migration ability of trophoblasts. HTR-8/SVneo cells were seeded into 6-well plates at a density of 1 × 10^6^ cells/well and cultured until 100% confluence to form a monolayer. A sterile 1 mL pipette tip was used to make a straight, uniform scratch perpendicular to the bottom of the well. Suspended cells were removed by gentle washing with pre-cooled PBS 3 times. To eliminate the interference of cell proliferation on wound healing, serum-free medium containing corresponding drugs was added to each well according to the grouping. Images of the same scratch position were captured under an inverted microscope at 0 h, 24 h and 48 h after scratching. The scratch width was measured using ImageJ software, and the cell migration rate was calculated.

### 2.8 Transwell migration assay

Transwell assay was performed to detect the vertical migration ability of trophoblasts. Cells were resuspended in serum-free medium and adjusted to a density of 5 × 10^5^ cells/mL. 200 μL of cell suspension was added to the upper chamber of the Transwell insert, with corresponding drugs added according to the grouping. 600 μL of complete medium containing 10% FBS was added to the lower chamber as a chemoattractant. After incubation for 24 h, the inserts were removed, and non-migrated cells on the upper surface of the membrane were gently wiped off with a sterile cotton swab. Cells on the lower surface were fixed with 4% paraformaldehyde for 30 min, stained with 0.1% crystal violet for 30 min, and washed 3 times with PBS. Images were captured from 5 random non-overlapping high-power fields (×200 magnification) under an inverted microscope. Cell counting was performed manually by two independent researchers in a blinded manner, with crystal violet-stained nuclei as the unified counting unit. For areas with partial cell overlap, visible individual nuclei were counted according to consistent criteria. The average cell number of the 5 fields was used for statistical analysis. The dense and overlapping appearance in the representative low-magnification image reflects the strong migration ability of cells; formal quantification was performed under high-power magnification with standardized counting rules. All experiments were independently repeated at least 3 times.

### 2.9 Flow cytometry analysis of cell cycle and apoptosis

#### 2.9.1 Cell cycle detection

After treatment according to the grouping, cells were collected, washed twice with pre-cooled PBS, and fixed with pre-cooled 70% ethanol at 4 °C overnight. The next day, cells were washed twice with PBS, incubated with 500 μL of PI/RNase A staining working solution in the dark at room temperature for 30 min. Cell cycle distribution was detected by flow cytometry, and the proportion of cells in G0/G1, S and G2/M phases was analyzed using ModFit LT software.

#### 2.9.2 Cell apoptosis detection

After treatment, cells were collected, washed twice with pre-cooled PBS, and resuspended in 100 μL of 1×Binding Buffer. 5 μL of Annexin V-FITC and 5 μL of PI staining solution were added to the suspension, mixed gently, and incubated in the dark at room temperature for 15 min 400 μL of 1×Binding Buffer was added to terminate the reaction, and the apoptosis rate was detected by flow cytometry within 1 h. The total apoptosis rate (early apoptosis + late apoptosis) was analyzed using FlowJo software.

### 2.10 Measurement of oxidative stress markers

#### 2.10.1 Intracellular ROS level detection

Fluorescence imaging assay (corresponding to Figures 2A,B): Cells were incubated with 10 μmol/L DCFH-DA probe at 37 °C for 20 min. After washing, cells were observed under a fluorescence microscope, and mean fluorescence intensity was quantified by ImageJ.

Flow cytometry assay (corresponding to Figures 2C,D): After probe incubation and washing, cells were digested and collected. Mean fluorescence intensity was detected by flow cytometry.2.10.2 Detection of MDA Content, GSH Content and SOD Activity.

After treatment, cells were lysed with RIPA lysis buffer containing protease inhibitors on ice for 30 min, and the supernatant was collected after centrifugation at 12,000 rpm for 15 min at 4 °C. The protein concentration was determined by BCA assay. The MDA content, GSH content and SOD activity in each sample were detected strictly according to the manufacturer’s instructions of the kits, and the results were normalized to the protein concentration of each sample.

### 2.11 Quantitative real-time PCR (RT-qPCR)

After treatment, total RNA was extracted from cells using TRIzol reagent, and the concentration and purity of RNA were detected by ultraviolet spectrophotometry (OD260/OD280 = 1.8–2.1 was considered qualified). 1 μg of total RNA was reverse transcribed into cDNA according to the instructions of the reverse transcription kit. RT-qPCR was performed on a real-time fluorescence quantitative PCR instrument using SYBR Green Mix kit. The reaction procedure was as follows: pre-denaturation at 95 °C for 30 s; 40 cycles of denaturation at 95 °C for 5 s, annealing and extension at 60 °C for 30 s β-actin was used as the internal reference gene, and the relative mRNA expression level of target genes was calculated using the 2^(-ΔΔCt)^ method. All primers were designed based on NCBI GenBank reference sequences and synthesized by Sangon Biotech Co., Ltd. (Shanghai, China). Their amplification specificity was verified by melting curve analysis during formal experiments, and all primer pairs exhibited a single sharp amplification peak with no non-specific products or primer dimers, ensuring the reliability of quantitative results. The primer sequences used in this study are shown in Table 1.

**TABLE 1:** Primer sequences for RT-qPCR.

Gene name	Forward primer (5'-3')	Reverse primer (5'-3')
*NFE2L2*(NRF2)	TCAGCGACGGAAAGAGTATGA	CCACTGGTTTCTGACTGGATGT
*HMOX1*(HO-1)	AAGACTGCGTTCCTGCTCAAC	AAAGCCCTACAGCAACTGTCG
*ACTB*(β-actin)	AGA​GCT​ACG​AGC​TGC​CTG​ACG	GTA​GTT​TCG​TGG​ATG​CCA​CAG​G

### 2.12 Western blot analysis

After treatment, total protein was extracted from cells using RIPA lysis buffer containing protease and phosphatase inhibitors. The protein concentration was determined by BCA assay. Equal amounts of protein (40 μg per lane) were separated by SDS-PAGE gel electrophoresis, and transferred to PVDF membranes. The membranes were blocked with 5% skim milk at room temperature for 2 h, then incubated with corresponding primary antibodies at 4 °C overnight. After washing 3 times with TBST (10 min each time), the membranes were incubated with HRP-conjugated secondary antibody at room temperature for 2 h. The protein bands were visualized using an ECL chemiluminescence detection system. The gray value of each band was analyzed using ImageJ software, with β-actin as the internal reference for total protein, and the corresponding total protein as the internal reference for phosphorylated protein. The relative expression level of each target protein was calculated.

### 2.13 Statistical analysis

All data were obtained from at least 3 independent repeated experiments and expressed as mean ± standard deviation (SD). Statistical analysis was performed using GraphPad Prism 9.0 software. For comparisons between two groups, unpaired t-test was used. For comparisons among three or more groups, one-way analysis of variance (ANOVA) followed by Tukey’s *post hoc* test was used. *P < 0.05 was considered statistically significant.*

## 3 Results

### 3.1 Quercetin attenuates H_2_O_2_-Induced injury, apoptosis, cell cycle disorder and migration dysfunction in HTR-8/SVneo cells

**FIGURE 1:**
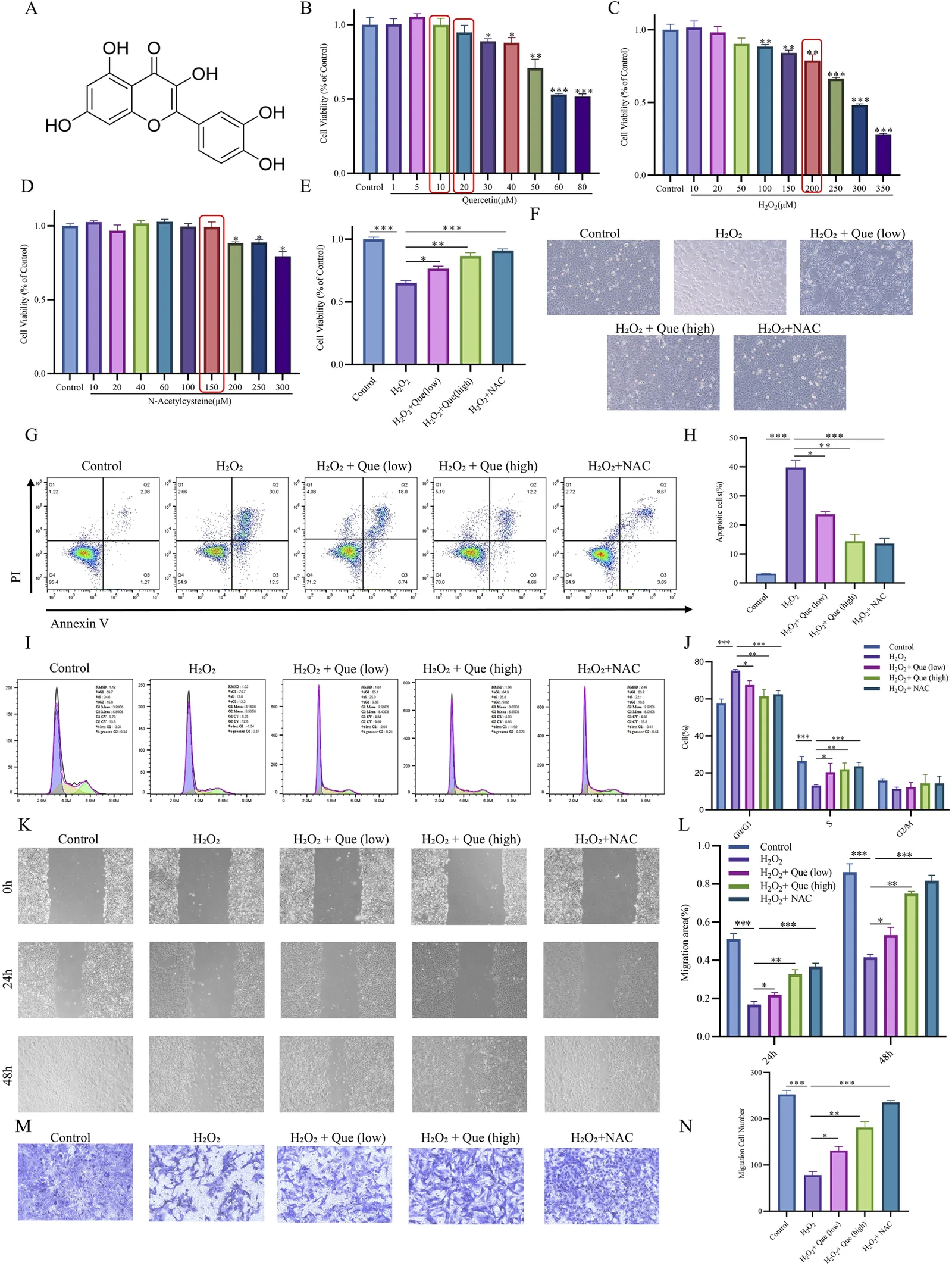
Quercetin attenuates H_2_O_2_-induced injury and dysfunction in HTR-8/SVneo cells **(A)** Chemical structure of quercetin. **(B–D)** CCK-8 assay for cell viability of HTR-8/SVneo cells treated with gradient concentrations of Que, H_2_O_2_ and NAC. **(E,F)** CCK-8 assay and morphological observation for the protective effect of Que on H_2_O_2_-injured cells. **(G,H)** Flow cytometry for cell apoptosis rate. **(I,J)** Flow cytometry for cell cycle distribution. **(K,L)** Wound healing assay for cell horizontal migration ability. **(M,N)** Transwell assay for cell vertical migration ability. All data are presented as mean ± SD from 3 independent biological replicates (n = 3). *P<0.05, **P<0.01, ***P<0.001.

In this study, CCK-8 assay was first performed to screen the safe administration concentration of quercetin, the optimal modeling concentration of H_2_O_2_ for oxidative damage, and the safe administration concentration of the positive control drug NAC. The chemical structure of quercetin is shown in Figure 1A.

Cell viability assay results showed that there was no significant difference in the viability of HTR-8/SVneo cells between the control group and groups treated with 1–20 μM quercetin, indicating no obvious cytotoxicity within this concentration range. Therefore, 10 μM quercetin was selected as the low-dose quercetin group (hereafter referred to as Que (low)) and 20 μM quercetin as the high-dose quercetin group (hereafter referred to as Que (high)) for subsequent intervention experiments (Figure 1B). H_2_O_2_ intervention reduced the viability of HTR-8/SVneo cells in a concentration-dependent manner, and the cell viability decreased to approximately 60% of the control group after intervention with 200 μM H_2_O_2_, which met the modeling requirements for oxidative damage model. Thus, 200 μM H_2_O_2_ was selected to establish the trophoblast oxidative damage model in subsequent experiments (Figure 1C). Intervention with 10–150 μM NAC had no significant effect on the viability of HTR-8/SVneo cells, so 150 μM NAC was selected as the positive control for subsequent experiments (Figure 1D).

Subsequently, 5 groups were set up to verify the protective effect of quercetin on trophoblast oxidative damage: control group, H_2_O_2_ model group, H_2_O_2_ + Que (low) group, H_2_O_2_ + Que (high) group, and H_2_O_2_ + NAC group. Cell viability results showed that compared with the control group, the cell viability of the H_2_O_2_ model group was significantly decreased (*P*<0.001). Compared with the H_2_O_2_ model group, Que (low) and Que (high) intervention significantly increased the viability of injured cells in a dose-dependent manner (*P*<0.01, *P*<0.001, respectively), and NAC also significantly reversed the H_2_O_2_-induced decrease in cell viability (*P*<0.001), with a protective effect similar to that of Que (high) (Figure 1E). Cell morphological observation showed that cells in the control group had good adherent growth status, regular morphology, and a spindle-shaped paving stone-like distribution; the number of cells in the H_2_O_2_ model group was significantly reduced, with obvious shrinkage and rounding. Que (low) and Que (high) intervention significantly ameliorated H_2_O_2_-induced abnormal cell morphology, and the cell morphology in the Que (high) group was closer to that of the control group; the NAC group also significantly restored the normal morphology of injured cells (Figure 1F).

To explore the effect of quercetin on H_2_O_2_-induced trophoblast apoptosis, flow cytometry was performed to detect the apoptosis rate of each group. The results showed that compared with the control group, the apoptosis rate of the H_2_O_2_ model group was significantly increased (*P*<0.001). Compared with the H_2_O_2_ model group, Que (low) and Que (high) intervention significantly reduced the cell apoptosis rate in a dose-dependent manner (*P*<0.05, *P*<0.001, respectively), and the NAC group also significantly inhibited H_2_O_2_-induced cell apoptosis (*P*<0.001) (Figures 1G,H).

Flow cytometry was further used to detect the cell cycle distribution of each group to explore the effect of quercetin on the cell cycle progression of injured cells. The results showed that compared with the control group, the proportion of cells in the G0/G1 phase was significantly increased (*P*<0.001) and the proportion of cells in the S phase was significantly decreased (*P*<0.001) in the H_2_O_2_ model group, indicating that H_2_O_2_ induced G0/G1 phase cell cycle arrest in HTR-8/SVneo cells. Compared with the H_2_O_2_ model group, Que (low) and Que (high) intervention significantly reversed H_2_O_2_-induced abnormal cell cycle distribution, reduced the proportion of cells in the G0/G1 phase, and restored the proportion of cells in the S and G2/M phases (*P*<0.01, *P*<0.001, respectively); the NAC group also significantly corrected the cell cycle arrest of injured cells (Figures 1I,J).

The migration ability of trophoblasts is the core biological function for normal placental development. In this study, wound healing assay and Transwell migration assay were performed to detect the ameliorative effect of quercetin on H_2_O_2_-induced trophoblast migration dysfunction. Wound healing assay results showed that compared with the control group, the 24 h and 48 h wound healing area of the H_2_O_2_ model group was significantly reduced (*P*<0.001), indicating that the migration ability was significantly inhibited. Compared with the H_2_O_2_ model group, Que (low) and Que (high) intervention significantly increased the 24 h and 48 h wound healing rate of cells in a dose-dependent manner (*P*<0.01, *P*<0.001, respectively), and the NAC group also significantly restored the migration and healing ability of injured cells (*P*<0.001) (Figures 1K,L). Transwell migration assay results were consistent with the trend of wound healing assay. Compared with the control group, the number of transmembrane cells in the H_2_O_2_ model group was significantly reduced (*P*<0.001). Compared with the H_2_O_2_ model group, Que (low) and Que (high) intervention significantly increased the number of transmembrane cells and ameliorated the transmembrane migration ability of cells in a dose-dependent manner (*P*<0.01, *P*<0.001, respectively); the NAC group also significantly reversed H_2_O_2_-induced cell migration dysfunction (Figures 1M,N).

The above results confirmed that quercetin can significantly ameliorate H_2_O_2_-induced oxidative damage in HTR-8/SVneo cells, inhibit cell apoptosis, correct G0/G1 phase cell cycle arrest, and effectively restore the migration function of trophoblasts. It has a clear trophoblast protective effect, which lays an experimental foundation for the subsequent exploration of its mechanism of action.

### 3.2 Quercetin inhibits H_2_O_2_-induced oxidative stress and activates the NRF2/HO-1 antioxidant pathway in HTR-8/SVneo cells

Oxidative stress is the core pathological mechanism of H_2_O_2_-induced trophoblast injury and dysfunction. To clarify the core biological effect and potential molecular mechanism of quercetin exerting the above trophoblast protective effect, DCFH-DA fluorescence staining, flow cytometry and biochemical kit detection were performed to measure the intracellular ROS level and key oxidation-antioxidant indexes of each group. Meanwhile, RT-qPCR and Western Blot were used to detect the mRNA and protein expression levels of key molecules in the NRF2/HO-1 pathway, the core regulatory pathway of oxidative stress.

**FIGURE 2:**
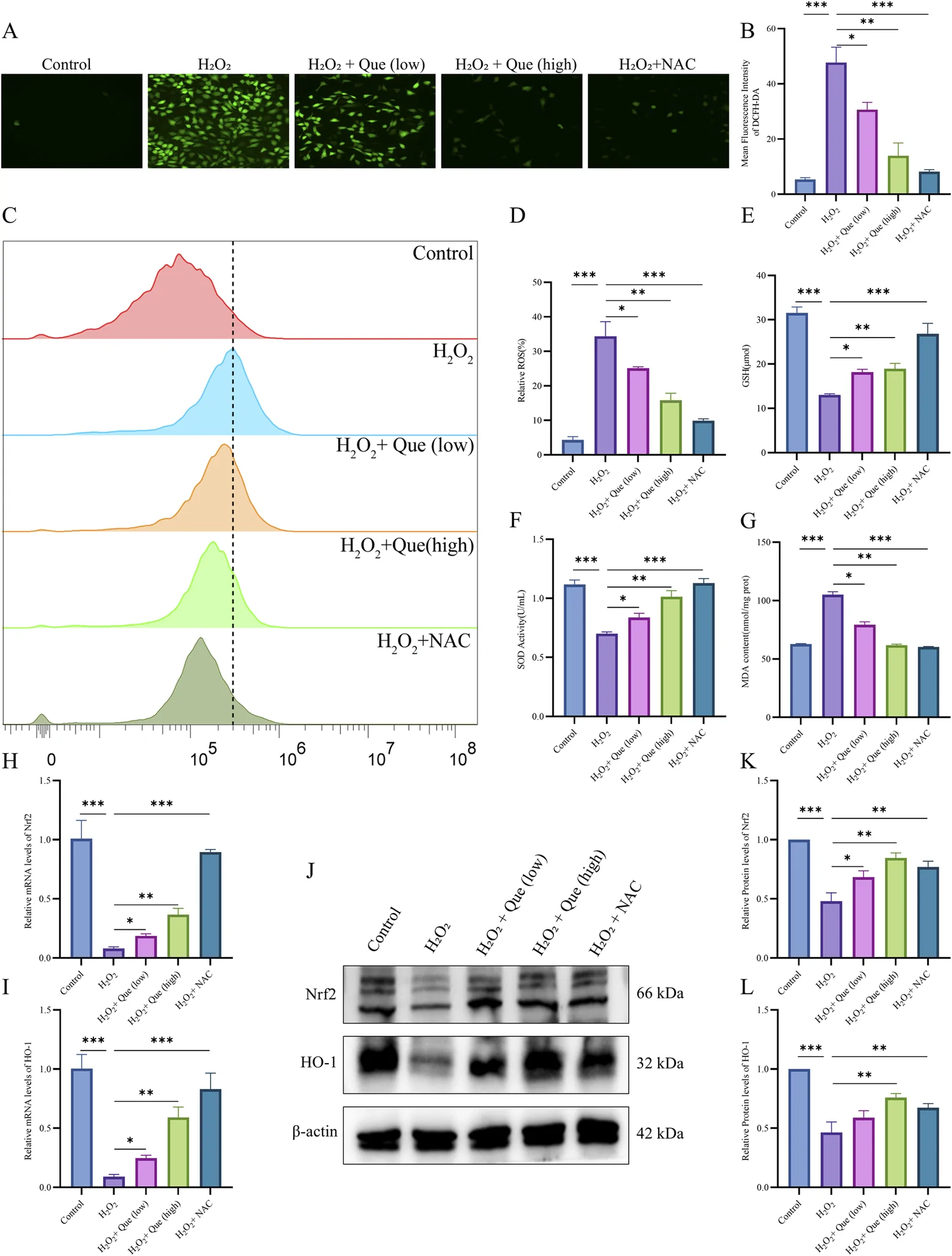
Quercetin suppresses oxidative stress and activates the NRF2/HO-1 pathway in HTR-8/SVneo cells. **(A,B)** Intracellular ROS level detected by DCFH-DA fluorescence imaging assay; **(C,D)** Intracellular ROS level detected by DCFH-DA flow cytometry assay. **(E–G)** Biochemical detection of SOD activity, GSH content and MDA content. **(H,I)** RT-qPCR for relative mRNA expression of *Nrf2* and *HO-1*. **(J–L)** Western Blot for protein expression of NRF2 and HO-1. All data are presented as mean ± SD from 3 independent biological replicates (n = 3). *P < 0.05, **P < 0.01, ***P < 0.001.

DCFH-DA fluorescence staining results showed that the green fluorescence signal in the control group was extremely weak, indicating that the intracellular ROS level was very low under basal conditions; the green fluorescence intensity in the H_2_O_2_ model group was significantly enhanced, indicating that H_2_O_2_ induced massive accumulation of intracellular ROS. After intervention with Que (low) and Que (high), the intracellular green fluorescence signal was significantly weakened in a dose-dependent manner, and the fluorescence signal in the NAC group basically restored to the level of the control group (Figures 2A,B; *P*<0.01, *P*<0.001, respectively). The results of intracellular ROS detection by flow cytometry further verified the above effect. The peak plot showed that the ROS fluorescence peak of the H_2_O_2_ model group was significantly shifted to the right compared with the control group, while the ROS fluorescence peaks of the Que (low) group, Que (high) group and NAC group were significantly shifted to the left compared with the H_2_O_2_ model group, and gradually restored to the level of the control group (Figures 2C,D).

Further detection of intracellular oxidative damage markers and endogenous antioxidant indexes was performed. The results showed that compared with the control group, the content of MDA, the end product of lipid peroxidation, was significantly increased (*P*<0.001), while the activity of endogenous antioxidant enzyme SOD and the content of core non-enzymatic antioxidant reduced GSH were significantly decreased in the H_2_O_2_ model group (*P*<0.001), indicating that H_2_O_2_ induced severe oxidative damage and imbalance of the endogenous antioxidant system in trophoblasts. Compared with the H_2_O_2_ model group, Que (low) and Que (high) intervention significantly reduced MDA content and upregulated SOD activity and GSH content in a dose-dependent manner (*P*<0.05, *P*<0.01, *P*<0.001, respectively); the NAC group also significantly reversed H_2_O_2_-induced oxidation-antioxidant system disorder (*P*<0.001) (Figures 2E–G).

The NRF2/HO-1 axis is the core classical pathway for the regulation of intracellular oxidative stress. To clarify the potential molecular mechanism of the antioxidant effect of quercetin, this study detected the mRNA and protein expression levels of key molecules NRF2 and HO-1 in this pathway. RT-qPCR results showed that compared with the control group, the mRNA expression levels of *Nrf2* and *HO-1* in the H_2_O_2_ model group were significantly downregulated (*P*<0.001). Compared with the H_2_O_2_ model group, Que (low) and Que (high) intervention significantly upregulated the mRNA expression of *Nrf2* and *HO-1* in a dose-dependent manner (*P*<0.01, *P*<0.001), and the NAC group also significantly increased the mRNA transcription levels of both genes (*P*<0.001) (Figures 2H,I). Western Blot results were completely consistent with the mRNA expression trend. Compared with the control group, the protein expression levels of NRF2 and HO-1 in the H_2_O_2_ model group were significantly decreased (*P*<0.001). Compared with the H_2_O_2_ model group, Que (low) and Que (high) intervention significantly upregulated the protein expression of NRF2 and HO-1 in a dose-dependent manner (*P*<0.01, *P*<0.001, respectively); the NAC group also significantly activated the NRF2/HO-1 pathway and upregulated the protein expression levels of both molecules (*P*<0.001) (Figures 2J–L).

The above results confirmed that quercetin can effectively eliminate excessive ROS in trophoblasts induced by H_2_O_2_, inhibit lipid peroxidation damage, and restore intracellular endogenous antioxidant capacity. Its antioxidant protective effect is closely related to the activation of the NRF2/HO-1 antioxidant pathway, which clarifies the core research direction for the subsequent screening of upstream regulatory targets and mechanism verification.

### 3.3 Screening of core targets and potential regulatory pathways of quercetin in regulating oxidative stress injury of trophoblasts via network pharmacology

**FIGURE 3:**
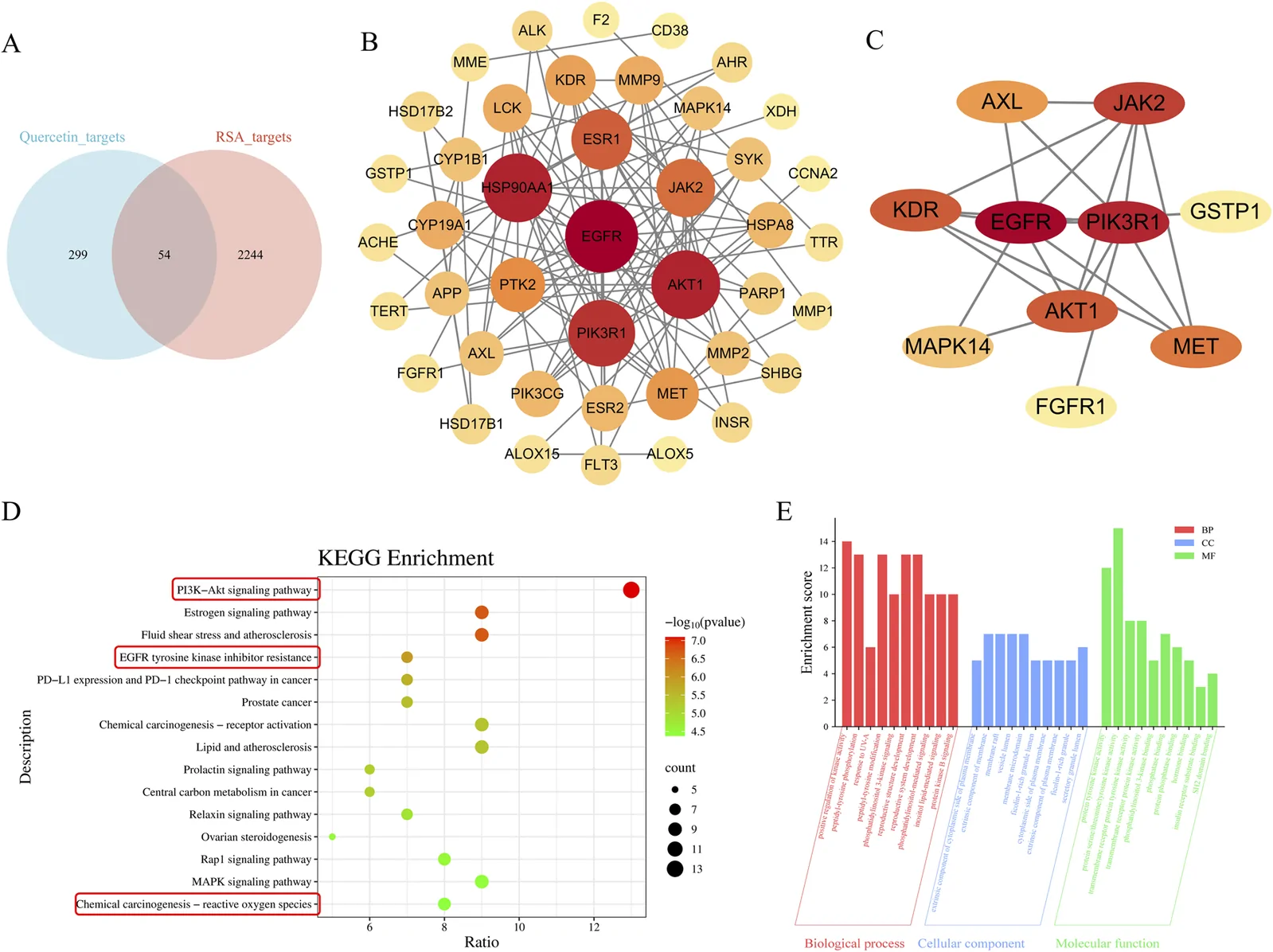
Network pharmacology screening of core targets and pathways of quercetin **(A)** Venn diagram of intersection targets between quercetin and recurrent spontaneous abortion-related disease targets. **(B,C)** PPI network and core module analysis of intersection targets. **(D)** KEGG pathway enrichment analysis of intersection targets. **(E)** GO functional enrichment analysis of intersection targets.

Based on the above results, we confirmed that quercetin exerts antioxidant protective effects on trophoblasts by activating the NRF2/HO-1 pathway, while its upstream regulatory mechanism remains unclear. Therefore, systematic screening was performed via network pharmacology. Venn diagram results showed that there were 54 intersection targets between the potential action targets of quercetin and the disease targets related to trophoblast oxidative damage, which were the potential core targets for quercetin to exert its effect (Figure 3A).

PPI network and module analysis results showed that EGFR, AKT1 and PIK3R1 (PI3K) were the core nodes in the network, forming a key regulatory module with EGFR as the upstream and PI3K/Akt as the core (Figures 3B,C). KEGG enrichment analysis results showed that the intersection targets were significantly enriched in the PI3K-Akt signaling pathway, EGFR tyrosine kinase regulatory pathway, reactive oxygen species-related pathway, etc., among which the PI3K-Akt pathway had the most significant enrichment degree (Figure 3D). GO functional enrichment analysis results showed that the intersection targets were mainly involved in biological processes such as cell proliferation and apoptosis, oxidative stress response, and cell migration, which were highly consistent with the biological effects of quercetin in this study (Figure 3E).

The above results provide bioinformatics theoretical support for the study on the upstream regulatory mechanism of quercetin, and identify the EGFR/PI3K/Akt pathway as the core research direction.

### 3.4 Verification of the binding affinity of quercetin to core targets via molecular docking and molecular dynamics simulation, and the activation effect of quercetin on the EGFR/PI3K/Akt pathway

**FIGURE 4:**
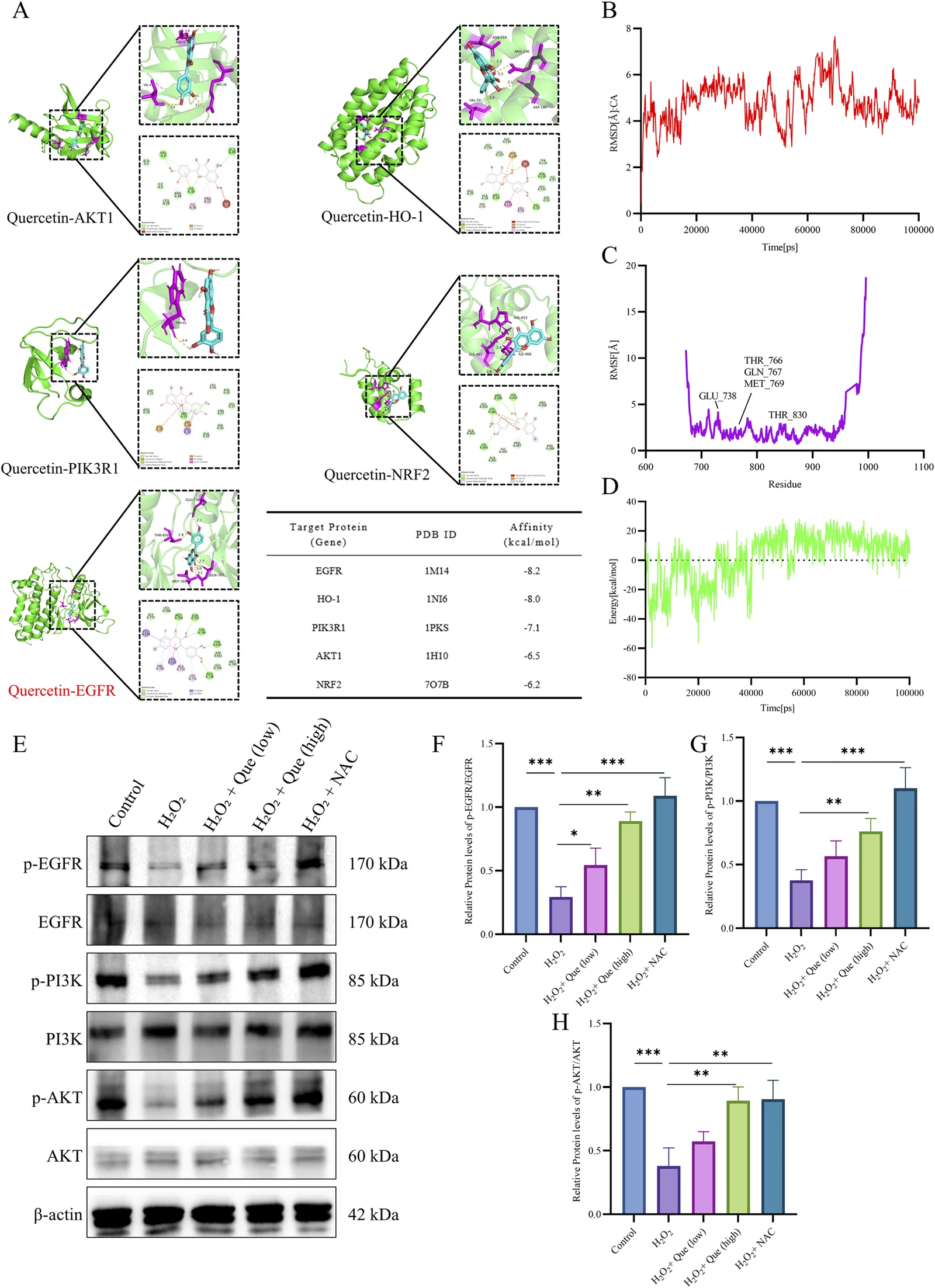
Molecular docking/dynamics validation and the activation effect of quercetin on the EGFR/PI3K/Akt pathway. **(A)** Molecular docking results: binding mode and binding free energy of quercetin with EGFR, AKT1, PI3K, NRF2 and HO-1. **(B–D)** 100 ns molecular dynamics simulation results of quercetin-EGFR complex: RMSD, RMSF and binding free energy curves. **(E–H)** WB for protein expression of p-EGFR, EGFR, p-PI3K, PI3K, p-AKT and AKT. All data are presented as mean ± SD from 3 independent biological replicates (n = 3). *P < 0.05, **P < 0.01, ***P < 0.001.

Based on the core targets screened by network pharmacology, molecular docking was performed to predict the binding potential and interaction mode of quercetin with EGFR, PI3K, AKT1, NRF2 and HO-1. The results showed that quercetin had good binding affinity to all the 5 core target proteins. Among them, the binding energy with EGFR was the lowest at −8.2 kcal/mol, and the binding energy with HO-1, PI3K, AKT1 and NRF2 was −8.0 kcal/mol, −7.1 kcal/mol, −6.5 kcal/mol and −6.2 kcal/mol, respectively, all of which were lower than the effective binding threshold of −5.0 kcal/mol. Three-dimensional binding mode and two-dimensional interaction analysis showed that quercetin could stably bind to the active pocket of each target protein through hydrogen bonds, hydrophobic interactions and other ways, forming a stable ligand-receptor complex (Figure 4A).

To further verify the binding stability of quercetin to the core target EGFR, 100 ns molecular dynamics simulation was performed in this study. The results showed that the RMSD value of the quercetin-EGFR complex gradually stabilized during the simulation, and the fluctuation amplitude was maintained within a reasonable range, indicating that the overall structure of the complex was stable. RMSF results showed that the amino acid residues in the binding pocket region of the complex had small fluctuation amplitude, indicating stable binding mode. The binding free energy results further confirmed that the binding of quercetin to EGFR maintained a stable low energy state throughout the simulation, verifying the reliability of the molecular docking results (Figures 4B–D).

On this basis, Western Blot was performed to detect the protein expression levels of key molecules in the EGFR/PI3K/Akt pathway in each group. The results showed that compared with the control group, the protein expression levels of p-EGFR, p-PI3K and p-AKT in the H_2_O_2_ model group were significantly downregulated (*P*<0.001), while there was no significant difference in the protein expression levels of total EGFR, PI3K and AKT. Compared with the H_2_O_2_ model group, Que (low) and Que (high) intervention significantly upregulated the protein expression of p-EGFR, p-PI3K and p-AKT in a dose-dependent manner (*P*<0.05, *P*<0.01, *P*<0.001, respectively); the NAC positive control group also significantly reversed H_2_O_2_-induced pathway inhibition and upregulated the expression levels of the above phosphorylated proteins (*P*<0.001) (Figures 4E–H).

The above results indicated that quercetin has potential binding affinity with core targets such as EGFR, and effectively activates the EGFR/PI3K/Akt signaling pathway in H_2_O_2_-injured trophoblasts, which clarifies the core regulatory axis for subsequent pathway verification experiments.

### 3.5 Inhibition of EGFR or NRF2 significantly reverses the proliferation protective and antioxidant effects of quercetin on HTR-8/SVneo cells

To further clarify the causal relationship rather than simple expression correlation of quercetin exerting trophoblast protective effect via the EGFR/PI3K/Akt-NRF2/HO-1 axis, intervention verification experiments were performed using gefitinib (EGFR-specific inhibitor) and ML385 (NRF2-specific inhibitor) to verify whether the protective effect of quercetin was reversed after blocking EGFR or NRF2.

**FIGURE 5:**
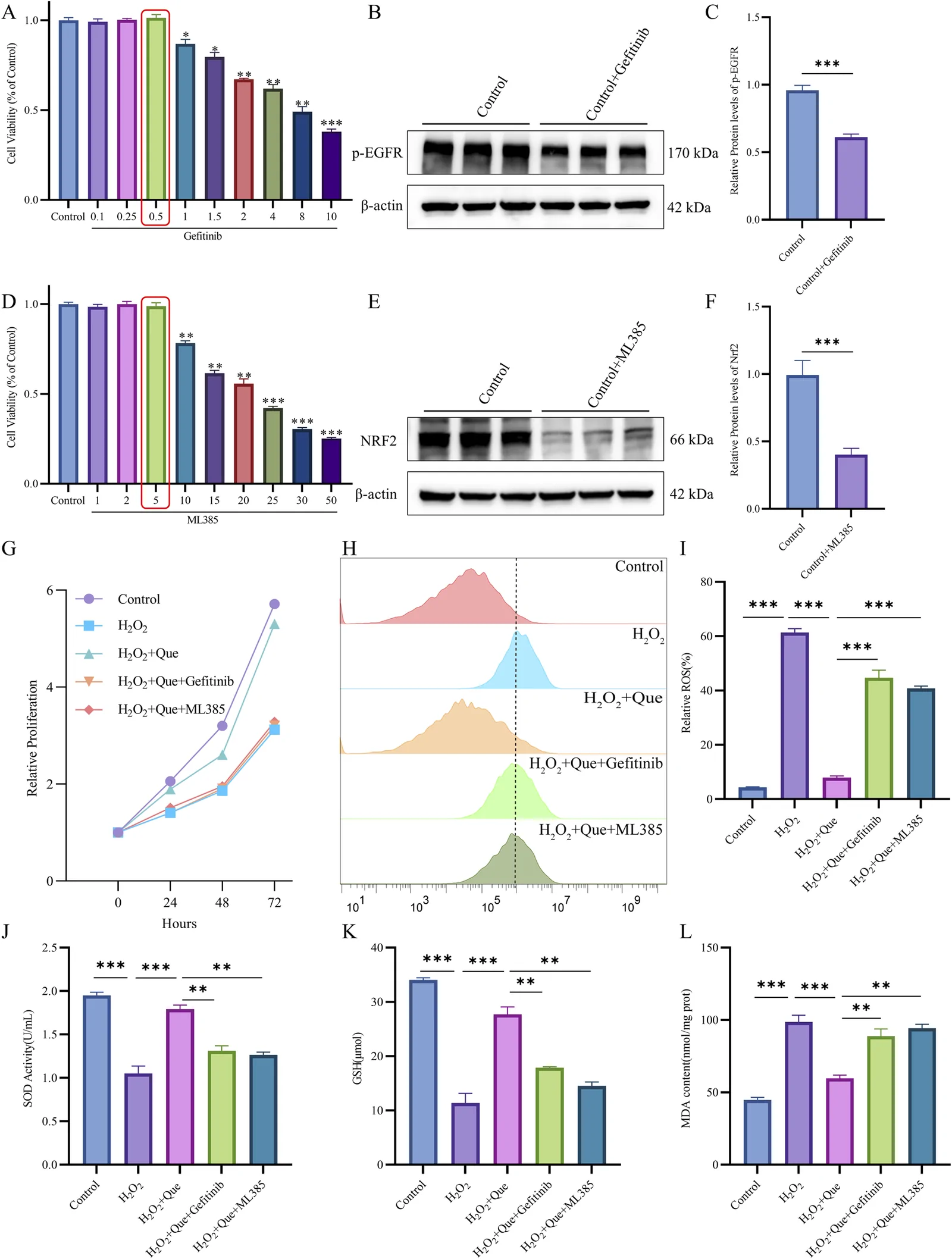
Inhibition of EGFR or NRF2 reverses the protective effects of quercetin. **(A,D)** CCK-8 assay for cell viability treated with gradient concentrations of gefitinib (EGFR inhibitor) and ML385 (NRF2 inhibitor). **(B,C, E,F)** WB validation of the inhibitory efficiency of gefitinib and ML385 on target proteins. **(G)** CCK-8 assay for cell proliferation curve of each group. **(H,I)** Flow cytometry for intracellular ROS level. **(J–L)** Biochemical detection of SOD activity, GSH content and MDA content. All data are presented as mean ± SD from 3 independent biological replicates (n = 3). *P<0.05, **P<0.01, ***P<0.001.

First, CCK-8 assay was performed to screen the safe administration concentration of the inhibitors. The results showed that there was no significant difference in the viability of HTR-8/SVneo cells between the control group and groups treated with 0.5 μM gefitinib or 5 μM ML385, indicating no obvious cytotoxicity. Therefore, these concentrations were selected for subsequent inhibitor intervention experiments (Figures 5A,D). Subsequently, the target inhibition efficiency of the inhibitors was verified. Western Blot results showed that intervention with 0.5 μM gefitinib significantly downregulated the protein expression level of p-EGFR in normal HTR-8/SVneo cells (*P*<0.001), confirming that it could effectively inhibit the phosphorylation and activation of EGFR (Figures 5B,C). Intervention with 5 μM ML385 significantly reduced the protein expression level of NRF2 in cells (P < 0.001), confirming that it could effectively inhibit the expression of NRF2 (Figures 5E,F). Both inhibitors had good target inhibition effects.On this basis, 5 groups were set up for pathway verification experiments: control group, H_2_O_2_ model group, H_2_O_2_ + Que (high) group, H_2_O_2_ + Que (high) + gefitinib group, and H_2_O_2_ + Que (high) + ML385 group. CCK-8 cell proliferation assay results showed that H_2_O_2_ significantly inhibited the proliferation ability of HTR-8/SVneo cells, while Que (high) intervention significantly reversed H_2_O_2_-induced proliferation inhibition. After intervention with gefitinib or ML385, the ameliorative effect of Que (high) on the proliferation ability of injured cells was significantly reversed, and the cell proliferation level was significantly lower than that of the H_2_O_2_ + Que (high) group (Figure 5G).

Flow cytometry results of intracellular ROS level showed that H_2_O_2_ induced massive accumulation of intracellular ROS, and Que (high) intervention significantly reduced excessive ROS. After intervention with gefitinib or ML385, the ROS-scavenging effect of Que (high) was significantly reversed, and intracellular ROS levels were markedly higher than those in the H_2_O_2_ + Que (high) group (P < 0.001) (Figures 5H,I). This difference from the results in Figure 2 is attributed to pathway inhibitor intervention, which blocks the EGFR/NRF2 axis and partially abolishes the antioxidant effect of quercetin, further verifying the pathway dependence of quercetin’s protective effect.Further detection of key intracellular oxidation-antioxidant indexes was performed. The results showed that Que (high) significantly reversed H_2_O_2_-induced decrease in SOD activity and GSH content, as well as increase in MDA content, and restored the intracellular endogenous antioxidant capacity. After intervention with gefitinib or ML385, the antioxidant protective effect of Que (high) was significantly inhibited, with significantly decreased SOD activity and GSH content, and significantly increased MDA content compared with the H_2_O_2_ + Que (high) group (*P*<0.01, *P*<0.001 respectively) (Figures 5J–L).

The above results confirmed that the proliferation protective and antioxidant effects of quercetin on H_2_O_2_-induced HTR-8/SVneo cells have clear EGFR and NRF2 pathway dependence, and blocking either EGFR or NRF2 can significantly reverse the protective effect of quercetin.

### 3.6 Inhibition of EGFR or NRF2 significantly reverses the ameliorative effect of quercetin on trophoblast migration function and the activation effect on the EGFR/PI3K/Akt-NRF2/HO-1 pathway

To further clarify the pathway dependence of quercetin ameliorating trophoblast migration function, as well as the upstream and downstream regulatory relationship between the EGFR/PI3K/Akt and NRF2/HO-1 pathways, wound healing assay was performed to verify the effect of inhibitor intervention on the migration protective effect of quercetin, and Western Blot was used to detect the protein expression levels of key molecules in the upstream and downstream pathways in each group.

**FIGURE 6:**
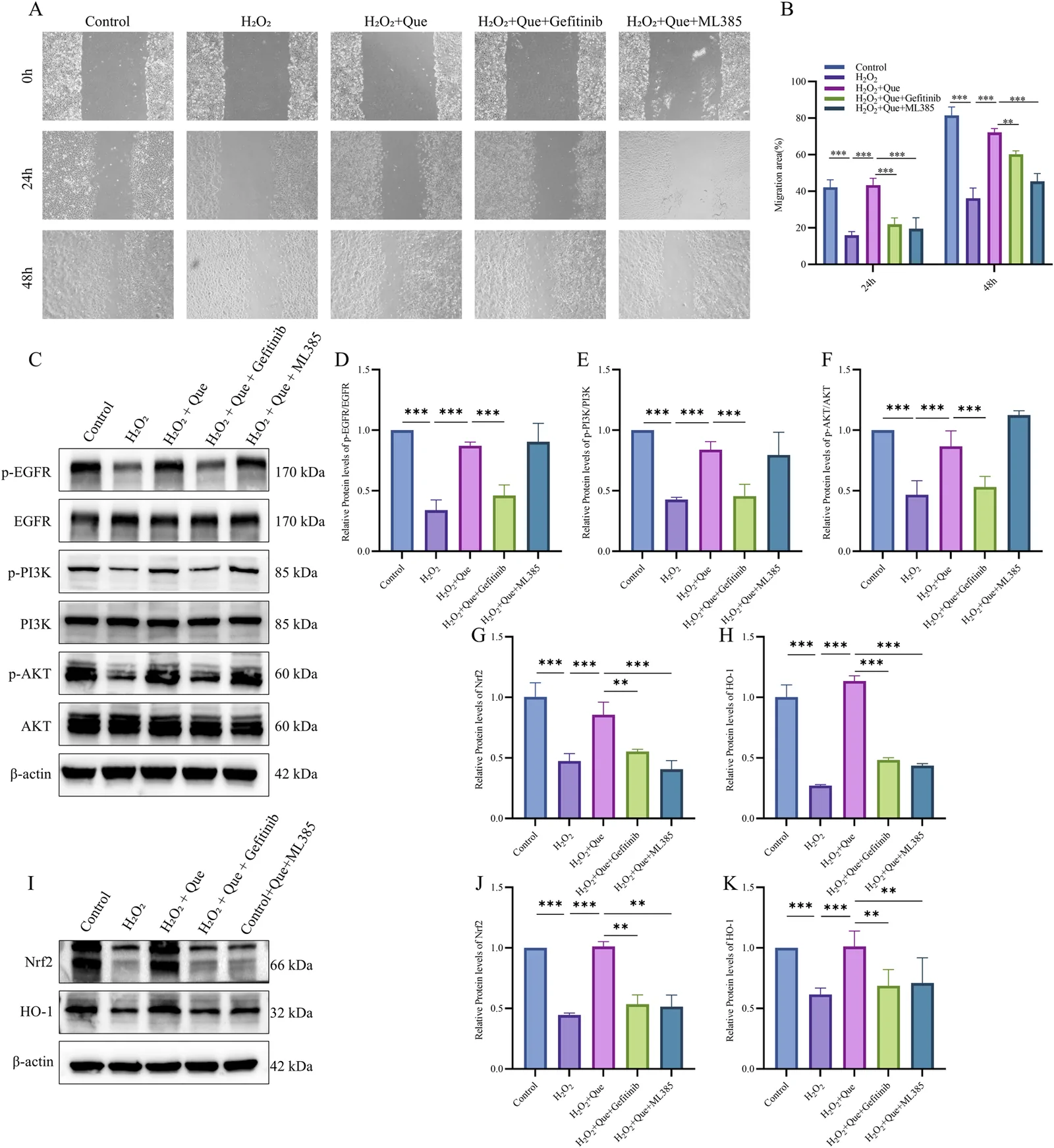
Inhibition of EGFR or NRF2 Reverses Quercetin-Induced Migration Improvement and Pathway Activation. **(A,B)** Wound healing assay for the migration ability of HTR-8/SVneo cells in each group, including representative scratch images (0 h, 24 h, 48 h) and quantitative statistics of migration rate. **(C–F)** Western blot (WB) for protein expression of p-EGFR, EGFR, p-PI3K, PI3K, p-AKT and AKT, with quantitative analysis of relative phosphorylation levels. **(G,H)** RT-qPCR for relative mRNA expression of *Nrf2* and *HO-1*. **(I–K)** WB for protein expression of NRF2 and HO-1, with quantitative analysis of relative expression levels. All data are presented as mean ± SD from 3 independent biological replicates (n = 3). **P < 0.01, ***P < 0.001.

Wound healing assay results showed that compared with the control group, the 24 h and 48 h wound healing rate of the H_2_O_2_ model group was significantly reduced, indicating that the migration ability was obviously inhibited. Compared with the H_2_O_2_ model group, Que (high) intervention significantly increased the 24 h and 48 h wound healing rate of cells, and effectively restored the migration ability of trophoblasts. After intervention with gefitinib or ML385, the ameliorative effect of Que (high) on trophoblast migration function was significantly reversed, and the 24 h and 48 h wound healing rate of cells was significantly lower than that of the H_2_O_2_ + Que (high) group (*P*<0.001) (Figures 6A,B).

Further detection of the protein expression levels of key molecules in the upstream and downstream pathways was performed. The results showed that compared with the control group, the protein expression levels of p-EGFR, p-PI3K and p-AKT in the H_2_O_2_ model group were significantly downregulated (*P*<0.001). Compared with the H_2_O_2_ model group, Que (high) intervention significantly upregulated the expression of the above phosphorylated proteins and activated the EGFR/PI3K/Akt pathway. After intervention with the EGFR inhibitor gefitinib, the upregulatory effect of Que (high) on p-EGFR, p-PI3K and p-AKT was significantly reversed (*P*<0.001), while intervention with the NRF2 inhibitor ML385 had no significant effect on Que (high)-induced phosphorylation and activation of EGFR, PI3K and AKT, confirming that NRF2 is the downstream regulatory target of the EGFR/PI3K/Akt pathway (Figures 6C–F).

For the downstream NRF2/HO-1 antioxidant pathway, we first verified its transcriptional changes via RT-qPCR: the results showed that compared with the control group, the mRNA expression levels of *Nrf2* and *HO-1* in the H_2_O_2_ model group were significantly decreased (*P*<0.001); compared with the H_2_O_2_ model group, Que (high) intervention significantly upregulated the mRNA expression of *Nrf2* and *HO-1* (*P*<0.001), which was significantly reversed by both the EGFR inhibitor gefitinib and the NRF2 inhibitor ML385 (*P*<0.01, *P*<0.001, respectively) (Figures 6G,H). Consistent with the transcriptional trends, the protein detection results of the NRF2/HO-1 pathway showed that compared with the control group, the protein expression levels of NRF2 and HO-1 in the H_2_O_2_ model group were significantly decreased (*P*<0.001); compared with the H_2_O_2_ model group, Que (high) intervention significantly upregulated the protein expression of NRF2 and HO-1 and activated this antioxidant pathway, and both gefitinib and ML385 significantly reversed the upregulatory effect of Que (high) on NRF2 and HO-1 (*P*<0.01, *P*<0.001, respectively) (Figures 6I–K). These results confirmed that the activation effect of quercetin on the NRF2/HO-1 pathway is dependent on the activation of upstream EGFR.

In summary, this study confirmed that quercetin may target and activate EGFR (predicted by molecular docking and molecular dynamics simulation), and then regulate the downstream PI3K/Akt-NRF2/HO-1 signaling axis, effectively eliminating excessive intracellular ROS in trophoblasts induced by H_2_O_2_, ameliorate oxidative stress damage, and finally exert a protective effect on H_2_O_2_-induced apoptosis, cell cycle arrest and migration dysfunction in HTR-8/SVneo cells. Blocking either upstream EGFR or downstream core effector molecule NRF2 can significantly reverse the cell protective effect and pathway activation effect of quercetin. This study clarifies the molecular mechanism of quercetin exerting trophoblast protective effect, and provides new potential targets and experimental basis for the prevention and treatment of placental oxidative stress-related diseases.

## 4 Discussion

This study is the first to demonstrate that quercetin, a natural flavonoid compound, ameliorates H_2_O_2_-induced oxidative stress injury and dysfunction in human trophoblast HTR-8/SVneo cells in a dose-dependent manner. Its core protective mechanism is dependent on the activation of the EGFR/PI3K/AKT signaling pathway, which further cascades to upregulate the downstream NRF2/HO-1 endogenous antioxidant defense axis, ultimately maintaining the proliferative homeostasis, anti-apoptotic capacity, migratory function, and cellular redox balance of trophoblasts. Our findings not only fill the gap in upstream and downstream mechanistic research of quercetin in the field of placental trophoblast protection, but also clarify the core targets and cascade regulatory relationship of its antioxidant effect, providing a novel theoretical basis and potential drug candidate for the prevention and treatment of placenta-derived APOs, including PE, FGR, and URSA.

The normal biological function of placental trophoblasts is the core basis for uterine spiral artery remodeling, sufficient placental perfusion, and the maintenance of a successful pregnancy. Trophoblast dysfunction mediated by excessive oxidative stress in the placental microenvironment is currently a well-recognized initiating pathological mechanism of placenta-derived APOs(Guo et al., 2023; Pintye et al., 2023; Yan et al., 2024). In this study, we established a trophoblast oxidative stress injury model using H_2_O_2_, which is a widely used cellular model for studying placental oxidative stress damage induced by ischemia-reperfusion during pregnancy. The results showed that quercetin significantly reduced intracellular ROS levels and the content of MDA, the end product of lipid peroxidation, in trophoblasts after H_2_O_2_ injury, while restoring the activity of the endogenous antioxidant enzyme SOD. Its antioxidant effect was comparable to that of the classic antioxidant positive control NAC, with a more pronounced protective effect observed at the high dose of quercetin. More importantly, we found that quercetin reversed H_2_O_2_-induced G0/G1 phase cell cycle arrest, increased apoptosis, and impaired migratory capacity in trophoblasts. These phenotypes are the core characteristics of trophoblast dysfunction, which directly determine the success of uterine spiral artery remodeling (Lapehn et al., 2025; Zhou et al., 2024). Previous studies have only preliminarily reported the antioxidant and organ-protective effects of quercetin in the reproductive system, but its protective effect on the core physiological functions of human trophoblasts and the direct causal relationship with oxidative stress have not been clarified (Dong et al., 2025; Li L. et al., 2024; Quan et al., 2023). Through multi-dimensional phenotypic validation, this study is the first to systematically confirm that quercetin comprehensively protects the core physiological functions of trophoblasts by antagonizing oxidative stress, laying a solid phenotypic foundation for the translational application of quercetin in obstetrics.

The NRF2/HO-1 axis is the core regulatory hub of the cellular endogenous antioxidant defense system, and also a key target for research on oxidative stress-related diseases (Li W. et al., 2024; Liu et al., 2024). Under physiological conditions, NRF2 is sequestered in the cytoplasm by Keap1and undergoes ubiquitination and proteasomal degradation. Upon oxidative stress, NRF2 dissociates from Keap1, translocates into the nucleus, and binds to the ARE to initiate the transcription of downstream antioxidant enzymes including HO-1, thereby scavenging excess ROS and maintaining cellular redox homeostasis (Adinolfi et al., 2023; Crisman et al., 2023; Tossetta et al., 2023). Existing clinical studies have confirmed that the expression of NRF2 and HO-1 is significantly downregulated in the placental tissues of patients with preeclampsia, accompanied by impaired antioxidant capacity of trophoblasts; whereas activation of the NRF2/HO-1 pathway significantly alleviates oxidative stress injury in trophoblasts (Chen et al., 2021; Qi Q. et al., 2025). Our results showed that quercetin significantly upregulated the mRNA and protein expression levels of NRF2 and HO-1 in trophoblasts after H_2_O_2_ injury, while the NRF2-specific inhibitor ML385 completely reversed the antioxidant effect and trophoblast function protection of quercetin. These findings directly confirm that the NRF2/HO-1 axis is an indispensable downstream effector target for quercetin to exert its trophoblast-protective effect. Although previous studies have verified the core role of the NRF2 pathway in trophoblast antioxidant defense (Luan et al., 2020), the upstream molecular mechanism by which quercetin regulates the NRF2/HO-1 axis in trophoblasts has not been elucidated, which is the core bottleneck limiting its target development.

To clarify the upstream regulatory mechanism of quercetin-mediated NRF2/HO-1 axis activation, we screened EGFR, PI3K, and AKT as the core functional targets of quercetin through network pharmacology analysis in this study. Molecular docking results further suggested that quercetin has favorable binding affinity with EGFR, PI3K, and AKT, suggesting that quercetin may activate these targets through potential binding. EGFR is a tyrosine kinase receptor widely expressed on the surface of trophoblast membranes, and its activation is a key upstream signal regulating the proliferation, migration, and anti-apoptotic capacity of trophoblasts (Chiang et al., 2024; Lin et al., 2024). Clinical studies have found that the expression and phosphorylation levels of EGFR are significantly reduced in the placental tissues of patients with preeclampsia (Karachrysafi et al., 2023; Xu et al., 2024). PI3K/AKT is the most core cascade pathway downstream of EGFR, and its activation depends on the phosphorylation modification of PI3K and AKT, rather than changes in total protein expression (Gu and Pang, 2025; Yang et al., 2025). Our results showed that H_2_O_2_ significantly inhibited the expression of EGFR and the phosphorylation levels of PI3K and AKT in trophoblasts, while quercetin intervention significantly reversed the abnormal changes of these indicators, confirming that quercetin can activate the EGFR/PI3K/AKT pathway. More critically, we clarified the upstream and downstream cascade regulatory relationship between this pathway and the NRF2/HO-1 axis for the first time through inhibitor reverse validation experiments: the EGFR-specific inhibitor gefitinib not only completely blocked the activating effect of quercetin on PI3K/AKT phosphorylation, but also significantly reversed the upregulation of the NRF2/HO-1 axis induced by quercetin, and completely abolished the antioxidant and trophoblast function protective effects of quercetin; in contrast, blocking NRF2 with ML385 only inhibited the expression of downstream HO-1, and had no effect on quercetin-induced upregulation of p-EGFR, p-PI3K, and p-AKT. These results clearly confirm that EGFR/PI3K/AKT is an essential upstream pathway for quercetin to activate the NRF2/HO-1 axis, and there is a clear cascade regulatory relationship between the two, rather than parallel independent pathways. Previous studies have confirmed that activated AKT can directly phosphorylate the Ser40 site of NRF2, prevent the binding of NRF2 to Keap1, and promote its nuclear translocation and transcriptional activity, which also provides a solid molecular mechanistic basis for our cascade regulation hypothesis (Zhang et al., 2023; Sen et al., 2025). This study is the first to verify that quercetin exerts a protective effect through the EGFR/PI3K/AKT-NRF2/HO-1 cascade signaling axis in human trophoblasts, and fully reveals the whole-chain molecular mechanism of quercetin in antioxidation and trophoblast function protection, which is the core innovation of this study.

From the perspective of clinical translation, there is still no effective etiological treatment for placenta-derived APOs in clinical practice at present, and the only definitive treatment for preeclampsia remains termination of pregnancy (Inthavong et al., 2024). Antioxidant therapy is currently the most promising direction for prevention and intervention. However, most synthetic antioxidants in previous studies have problems such as insufficient safety during pregnancy and poor target specificity, making it difficult to apply to pregnant populations in clinical practice. Quercetin is a natural flavonoid widely present in daily fruits, vegetables and medicinal plants, with good biosafety and oral bioavailability (Georgiou et al., 2023). A number of studies have confirmed its safety during pregnancy exposure, and it has the potential to be used as a dietary supplement or preventive drug during pregnancy (Delenko et al., 2024; Shi et al., 2025). This study systematically elucidates the protective effect and molecular mechanism of quercetin on trophoblasts, providing sufficient theoretical support for its subsequent preclinical research and clinical translation.

Notably, the 10–20 μM concentrations of quercetin used in this *in vitro* pharmacological intervention study are higher than the peak free plasma concentrations after conventional oral administration. This is a common setting for cellular mechanistic studies, and the *in vivo* efficacy needs to be further verified with optimized delivery regimens to improve bioavailability.

In addition, the regulatory effect of quercetin on the EGFR/PI3K/AKT axis is highly context-dependent. In tumor cells with aberrantly hyperactivated EGFR, quercetin exerts an inhibitory effect on abnormal pathway activation to suppress malignant proliferation; in normal trophoblast cells with physiological EGFR expression, quercetin activates the pathway to exert cytoprotective effects against oxidative damage. These two phenomena are observed in completely different pathophysiological contexts and do not contradict each other.

There are still some limitations in this study. First, all experiments were performed only *in vitro* using the HTR-8/SVneo immortalized trophoblast cell line. As this cell line is immortalized by SV40 large T antigen, its baseline EGFR expression and PI3K/AKT signaling activity may differ from those of primary human extravillous trophoblasts. The reliability of the conclusions needs to be further validated in primary cells and placental organoid models. Second, the acute H_2_O_2_-induced injury model adopted in this study can recapitulate the core process of trophoblast oxidative damage, but cannot fully replicate the chronic and progressive oxidative stress microenvironment in placenta-derived adverse pregnancy outcomes such as preeclampsia. More clinically relevant *in vivo* models will be applied in our follow-up research. Third, this study only evaluated the total protein and mRNA levels of NRF2 to reflect the activation of the antioxidant axis, but did not directly detect the nuclear translocation of NRF2, which is a critical step for its transcriptional function. We will further verify the regulatory effect of quercetin on NRF2 nuclear translocation via nuclear-cytoplasmic fractionation and immunofluorescence confocal imaging in subsequent work. Fourth, the present study mainly focused on the EGFR/PI3K/AKT-NRF2/HO-1 signaling axis, while quercetin may exert cytoprotective effects through multiple pathways such as anti-inflammation, endoplasmic reticulum stress regulation and mitochondrial function maintenance. The *in vivo* protective efficacy and pregnancy safety of quercetin also remain to be verified in animal models, which is a critical step for further clinical translation.

In conclusion, this study confirms that quercetin can upregulate the downstream NRF2/HO-1 antioxidant defense axis by activating the EGFR/PI3K/AKT signaling pathway, thereby inhibiting oxidative stress injury and improving the proliferation, anti-apoptosis and migration functions of human trophoblasts. This study not only reveals a novel molecular mechanism of quercetin in protecting trophoblasts, but also provides a new potential target and natural candidate drug for the prevention and treatment of placenta-derived adverse pregnancy outcomes.

## Data Availability

The datasets presented in this study can be found in online repositories. The names of the repository/repositories and accession number(s) can be found in the article/Supplementary Material.
